# Radiological Imaging in Gastroparesis: A Narrative Literature Review of Diagnostic and Evaluation Techniques

**DOI:** 10.7759/cureus.110107

**Published:** 2026-06-02

**Authors:** Murtaza H Raouf, Muhammad Raouf, Fahed Youssef

**Affiliations:** 1 Respiratory Medicine, Ipswich Hospital, East Suffolk and North Essex NHS Foundation Trust (ESNEFT), Ipswich, GBR; 2 School of Clinical Medicine, University of Cambridge, Cambridge, GBR; 3 General Surgery, Ipswich Hospital, East Suffolk and North Essex NHS Foundation Trust (ESNEFT), Ipswich, GBR

**Keywords:** barium study, gastric emptying, gastric emptying scintigraphy (ges), gastric motility, gastroparesis, radiological imaging, ultrasound

## Abstract

Gastroparesis is a gastrointestinal disorder characterised by delayed gastric emptying without mechanical obstruction, leading to symptoms such as nausea, vomiting, bloating, and epigastric discomfort. Accurate diagnosis and evaluation of this condition are critical, as untreated gastroparesis can result in complications including malnutrition, electrolyte imbalances, and mucosal injury. Radiological imaging plays a central role in both diagnosing and assessing the severity of gastroparesis. This narrative review provides an overview of current imaging modalities, including gastric emptying scintigraphy (GES), barium studies, gastric emptying of radiopaque markers, transabdominal ultrasound, and magnetic resonance imaging (MRI). GES remains the gold-standard diagnostic tool due to its quantitative assessment of gastric emptying, though limitations such as cost, time requirements, radiation exposure, and limited accessibility may restrict its use. Alternative modalities, including ultrasound and MRI, offer noninvasive, safer, and increasingly accurate options, with MRI showing promising correlations with GES in measuring gastric motility and emptying. Barium studies and radiopaque markers play complementary roles in ruling out obstruction or providing screening. Understanding the strengths and limitations of these imaging techniques can guide clinicians in selecting appropriate diagnostic strategies and improve patient management in gastroparesis.

## Introduction and background

Gastroparesis is defined as a gastric motility disorder that results in delayed gastric emptying without the presence of a mechanical obstruction. Patients commonly present with symptoms such as nausea, vomiting, early and postprandial fullness, bloating, and epigastric discomfort. Complications such as malnutrition, electrolyte imbalances, oesophagitis, and Mallory-Weiss tears may develop if this condition is left untreated [1].

Common causes of gastroparesis include diabetes, previous upper gastrointestinal surgery (particularly where there may be surgical injury to the vagus nerve), and certain medications known to delay gastrointestinal motility, such as opioids. However, the exact pathophysiology of gastroparesis is still not fully understood. Some theories have been suggested, including pyloric dysfunction, neuromuscular abnormalities, and autonomic neuropathy.

Diagnosis of gastroparesis is not straightforward and requires a multi-step approach. A common approach is to first rule out structural causes such as mechanical obstruction. Once mechanical obstruction has been excluded, delayed gastric emptying must be demonstrated objectively, i.e., retention of food in the stomach exceeding expected times after standard test meals (usually assessed via gastric emptying scintigraphy (GES)) [2].

This distinction is highly important, as gastroparesis symptoms can often overlap with other conditions such as functional dyspepsia (where gastric emptying is normal) or mechanical obstruction (where motility is unaffected but a physical blockage is present).

Hence, imaging assumes an important role in the diagnosis of gastroparesis, with particular focus on GES, magnetic resonance imaging (MRI), ultrasound, barium studies, and radiopaque marker techniques. These techniques can vary in their approach but ultimately aim to support the diagnostic process. Some techniques focus on ruling out obstruction, while others assess the time taken for gastric emptying or evaluate motility patterns.

This narrative literature review aims to provide a practical overview of the imaging modalities used in evaluating suspected gastroparesis. This article discusses the clinical applications, advantages, and limitations of GES, barium studies, radiopaque marker studies, ultrasound, and MRI. It aims to provide a practical overview of these modalities in the assessment of gastrointestinal motility. This work is not intended to be a systematic review, and the discussion is therefore selective rather than exhaustive. 

## Review

Methodology 

A literature search was conducted using PubMed, employing the following keywords: “gastroparesis MRI,” “gastroparesis ultrasound,” “gastroparesis gastric emptying scintigraphy,” “gastroparesis barium,” and “gastroparesis radiopaque markers.” Priority was given to literature published between 2020 and 2026 to ensure relevance to current clinical practice. However, earlier studies were included where they provided important context, supported key arguments, or reported particularly notable findings.

Since the purpose of this paper is to offer an overview of imaging modalities rather than a comprehensive systematic review, a formal review methodology was not used.

GES 

Gastro-oesophageal scintigraphy is widely considered the gold standard investigation for the diagnosis of gastroparesis. This technique enables direct, quantitative assessment of gastric emptying by tracking a radiolabelled meal through the stomach over time.

GES assesses gastric emptying by tracking the rate at which a radiolabelled meal exits the stomach. The standardisation of this test has been supported by consensus guidelines [2].

Protocols have been established, including the composition of the meal (typically technetium-99m-labelled egg whites), imaging intervals, and time benchmarks over a four-hour period. Such standardisation improves consistency and makes it easier to compare results across different institutions.

GES is usually not first-line in many healthcare systems due to cost and resource demands, i.e., limited nuclear medicine facilities. However, its diagnostic accuracy makes it highly valuable. The test involves serial imaging at regular intervals (commonly at 0, 1, 2, and 4 hours post ingestion), during which radioactivity within the stomach is measured. The percentage of meal retention at each time point is compared against established normal values. The quantitative approach of GES is advantageous, as it provides a measurable target that can be used to monitor response to treatment.

Some argue that despite these advantages, GES has a few limitations. Strict preparation is required to ensure diagnostic reliability. Patients must fast appropriately, and diabetic patients must ensure adequate glycaemic control prior to the test, as hyperglycaemia itself can transiently delay gastric emptying and therefore lead to false-positive results. Furthermore, the meal must be consumed in full within a specified time frame (typically 10 minutes). These requirements can be difficult for patients with severe nausea or early satiety, which may reduce compliance and affect the accuracy of the results. 

Another practical limitation is the prolonged nature of the test. This becomes particularly problematic in acute or critical care settings, where four hours to complete the study may not be possible [3]. In these situations, clinicians often have to use other imaging methods, even though they may not be considered as well validated.

Many believe that radiation exposure is also important to consider, particularly in younger patients and pregnant women.

MRI 

MRI has increasingly been explored as an alternative technique for assessing gastric motility and gastric emptying. One of the main advantages of MRI is that it does not involve exposure to ionising radiation. In addition, MRI provides excellent soft tissue contrast and allows visualisation of both soft tissues, as well as gas, solids, and liquids across different organs, along with the functional aspects of the gastrointestinal tract. 

MRI can often demonstrate the ability to evaluate the entire gastrointestinal tract and can evaluate both structural and functional abnormalities as was shown in this case report [4], which also showed and reinforced that MRI is capable of assessing several parts of the gastrointestinal tract via a single test. 

Unlike scintigraphy, MRI can provide detailed information about gastric morphology and intragastric volume distribution. Several studies have demonstrated that MRI can generate three-dimensional (3D) reconstructions of the stomach, allowing investigators to examine changes in gastric volume, surface area, and wall tension during the process of gastric emptying. MRI has also been used to evaluate gastric motility, including details of changes in volume, surface areas, and wall tension distribution in various gastric compartments. These measurements may offer insights into the physiological mechanisms possibly causing gastroparesis [5].

One can argue that MRI has shown the ability to assess the relative contributions of different gastric compartments in facilitating gastric emptying during and after a meal, with noted variations in volumes by region. An objective description of gastric morphology during gastric emptying has been documented via MRI, which might not have been obtainable with alternative modalities [6].

Another study, albeit done on mice, showed that MRI could reproducibly detect increased gastric volume following ingestion of a standard meal which progressively decreased and that the gastric volume at a particular time was higher in mice with diabetic gastroparesis compared to the normal group [7].

Parameters such as peristaltic wave velocity and gastric content distribution have been assessed using dynamic MRI sequences. In one study examining lung transplant recipients [8], MRI-derived measurements of gastric motility were found to correlate with gastric retention parameters obtained from scintigraphy. Reduced peristaltic wave velocity was associated with prolonged gastric emptying times, suggesting that MRI measurements may reflect clinically relevant functional abnormalities.

As seen in a comparative study between different modalities, where imaging results from MRI and GES were compared in a group of lung transplant recipients [8], the parameters assessed were different due to the nature of the modalities. However, the study aimed to determine whether there was any correlation between these measured parameters.

Peristaltic wave speed on MRI was lower in patients who had slower stomach emptying. In other words, this implied a link between reduced velocity and prolonged gastric emptying. Interestingly, a significant positive correlation was also observed between gastric content volume ratios (MRI) and retention rate, as well as gastric emptying time.

The study also found that individuals with larger amounts of stomach contents on MRI early in the study tended to have slower emptying later.

Overall, these findings suggest that MRI measurements of stomach movement and emptying correlate well with the standard gastric emptying test (GES). This indicates that MRI could be a useful alternative method for assessing gastric emptying.

A study by Ajaj et al. [9] investigated whether real-time MRI could differentiate between patients with motility disorders and healthy individuals. They found that patients with gastroparesis had reduced gastric motility compared to healthy volunteers.

MRI was shown to be a reliable method for measuring this difference and clearly distinguished between the two groups based on motility scores.

The study also demonstrated that, following treatment, motility scores in patients with gastroparesis improved on MRI. This suggests that MRI could be useful not only for diagnosis but also for monitoring response to treatment, particularly as it is non-invasive and can be repeated easily.

However, MRI has several limitations. Its availability is limited due to the high cost of equipment. Running the scan is also expensive and time-consuming. Patients may experience claustrophobia and must remain still for the duration of the scan, which can be uncomfortable.

While MRI shows promise, it should be noted that protocols for diagnosing gastroparesis using MRI are not yet standardised.

Ultrasound 

Ultrasound has also been considered as a potential investigative tool for assessing gastric emptying, motility, and diagnosing gastroparesis. It offers several practical advantages, including widespread availability, relatively low cost, and the absence of ionising radiation. These characteristics make ultrasound a valuable option, particularly in settings where access to more advanced imaging modalities may be limited. 

Conventional two-dimensional (2D) ultrasound techniques can be used to measure gastric dimensions following ingestion of a test meal. Serial measurements allow estimation of gastric emptying rates based on changes in gastric volume over time. 

More recently, advances in ultrasound technology have expanded its potential applications. 3D ultrasound is a relatively recent development that enables more accurate measurement of organ mass and volume, making it useful for assessing gastric volume. Compared to conventional 2D ultrasound, it is less limited by the shape of the structure being examined, as it typically uses a matrix transducer to capture volumetric data. This is particularly beneficial when evaluating the stomach, whose irregular shape can lead to measurement inaccuracies with 2D ultrasonography [10]. It has been shown that 3D ultrasound can distinguish between healthy and gastroparetic patients with good diagnostic performance [10]. 

A recent advancement in ultrasound technology is high-frame-rate peristaltic analysis, which enables real-time, non-invasive, and quantitative assessment of gastric motility [11]. Using a dedicated peristaltic analyser, this technique provides enhanced temporal resolution and motion sensitivity compared with conventional ultrasound, allowing precise tracking of peristaltic activity. It can help in objective evaluation of parameters such as peristaltic velocity, propagation distance, contraction frequency, and contraction intensity. The combination of visualisation and quantification can improve detection of abnormal motility patterns and offers the potential for greater standardisation in gastric motility assessment. 

However, its clinical applicability remains to be fully established. Diagnostic accuracy requires further validation in clinical populations, and the technique depends on specialised software and hardware. As with all ultrasound-based modalities, it is operator-dependent, and image quality may be affected by patient-related factors such as body habitus and the presence of bowel gas. This represents a limitation compared with MRI, which more reliably differentiates between gas, solids, and liquids. 

Studies have shown that ultrasound can be useful in assessing gastric motility in diabetic gastroparesis, particularly when combined with meal-based testing. Serial measurements can detect delayed gastric emptying, a key feature of the condition. For example, a slower reduction in proximal gastric size following a meal has been observed in patients with gastroparesis compared with healthy controls, which is consistent with impaired gastric emptying. Although delayed emptying reflects only one aspect of gastroparesis pathophysiology, it remains a widely used clinical marker [12].

Transabdominal ultrasound (TUS) has been evaluated as a potential alternative to GES, the current gold standard. One study [13] addressed the physiological differences in gastric handling of solids and liquids, where solid and semi-solid emptying is primarily mediated by peristaltic activity, and liquid emptying by pressure gradients, by using a standardised cellulose-based semi-solid meal to improve measurement consistency. 

For solids and semisolid meals, emptying is largely under the influence of a peristaltic pump. For liquids, a pressure pump mechanism is involved [14].

The results demonstrated no significant differences in overall gastric emptying curves between scintigraphy and ultrasound-based assessment (TUS-OSCA). The 50% gastric emptying times were comparable (89.4 ± 1.8 minutes for scintigraphy vs 92.5 ± 1.7 minutes for TUS-OSCA), with a strong correlation (r = 0.922, p < 0.001). These findings suggest that TUS-OSCA may represent an accurate, non-invasive alternative for evaluating gastric emptying. 

Nonetheless, ultrasound remains limited in assessing gastric emptying following solid meals [15]. This is clinically important, as delayed emptying of solids is often more pronounced than that of liquids in gastric motility disorders. Therefore, optimal diagnostic approaches should ideally include accurate assessment of solid-phase gastric emptying. 

Barium and radiopaque markers 

Barium studies have been used historically but are now considered a more outdated technique. Because of this, barium does not accurately reflect how the stomach processes and empties a typical meal under physiological conditions. However, barium studies remain useful for outlining gastric structure and excluding mechanical obstruction, which is an important step in evaluating conditions such as gastroparesis. 

Gastroparesis can present with several features on barium studies, such as absent or reduced gastric peristalsis. This may appear as proximal gastric dilatation, delayed emptying of barium, or the presence of residual fluid or debris in the stomach. However, these findings are difficult to quantify accurately, as it is challenging to determine how much barium has emptied over time. As a result, barium studies generally provide qualitative or semi-quantitative assessments rather than precise measurements. Thus, GES has mostly replaced it for this reason [16]. 

Barium is chemically inert, meaning it does not behave like real food once it enters the stomach. Studies show that, unlike a normal meal, gastric motility and emptying are influenced by factors such as nutrient composition, caloric content, and feedback from the small intestine. Therefore, barium does not replicate these properties [17]. 

Barium studies are also not standardised, meaning results can vary significantly. They depend on factors such as patient positioning, gastric contents, and timing of imaging, as well as the experience of the person interpreting the images. This affects reproducibility. 

Radiopaque markers are inexpensive and widely available, but they also have limitations. The quality of the images depends on the skill of the radiographer. Additionally, X-ray quality may be reduced in certain patients, such as those who are overweight, due to poorer X-ray penetration. Even when good-quality images are obtained, interpretation can be challenging. For example, variations in patient anatomy or markers overlapping with structures such as the spine can make them difficult to identify and assess [18]. A comparison of the different imaging modalities is presented in Table 1.

**Table 1:** Imaging modalities used in gastroparesis evaluation

Modality	Primary role	Advantages	Limitations
Gastric emptying scintigraphy (GES)	Quantitative assessment of gastric emptying	Gold standard, standardised protocols, objective measurements	Radiation exposure, lengthy test duration, limited availability
MRI	Assessment of gastric motility, volume, and morphology	No radiation, high soft-tissue contrast, functional imaging potential	Expensive, limited availability, lack of standardised protocols
Ultrasound	Assessment of gastric volume and motility	Widely available, inexpensive, non-invasive	Operator dependent, affected by gas and body habitus
Barium studies	Exclusion of structural obstruction	Widely available, useful anatomical overview	Poor quantitative assessment of gastric emptying
Radiopaque marker studies	Assessment of gastrointestinal transit	Inexpensive and accessible	Limited validation for gastric emptying, image interpretation challenges

## Conclusions

In summary, this discussion has outlined what gastroparesis is and briefly described how it is diagnosed. Several investigation methods have been covered, including GES, barium studies, radiopaque markers, ultrasound, and MRI, along with their advantages and limitations. 

GES remains the gold standard test due to its ability to provide quantitative assessment of gastric emptying. However, other imaging modalities may be useful in certain clinical situations, particularly in excluding structural causes such as obstruction. Nevertheless, factors such as cost, availability, and lack of standardised protocols currently limit the routine use of these alternative techniques in clinical practice. Further research is likely to clarify the role of emerging imaging techniques in the evaluation of gastroparesis and may ultimately lead to improved diagnostic strategies in the future. 
